# Glutamate Cysteine Ligase—Modulatory Subunit Knockout Mouse Shows Normal Insulin Sensitivity but Reduced Liver Glycogen Storage

**DOI:** 10.3389/fphys.2016.00142

**Published:** 2016-04-21

**Authors:** Suzie Lavoie, Pascal Steullet, Anita Kulak, Frederic Preitner, Kim Q. Do, Pierre J. Magistretti

**Affiliations:** ^1^Department of Psychiatry, Centre for Psychiatric Neuroscience, Lausanne University Hospital and University of LausanneLausanne-Prilly, Switzerland; ^2^Orygen, The National Centre of Excellence in Youth Mental Health, Centre for Youth Mental Health, The University of MelbourneParkville, VIC, Australia; ^3^Mouse Metabolic Evaluation Facility, Center for Integrative Genomics, University of LausanneLausanne, Switzerland; ^4^Laboratory of Neuroenergetics and Cellular Dynamics, Brain Mind Institute, Ecole Polytechnique Fédérale de LausanneLausanne, Switzerland; ^5^BESE Division, King Abdullah University of Sciences and Technology (KAUST)Thuwal, Saudi Arabia

**Keywords:** glutathione, GCLM knockout, glycogen, insulin, glycemia, resident-intruder stress, cortisol

## Abstract

Glutathione (GSH) deficits have been observed in several mental or degenerative illness, and so has the metabolic syndrome. The impact of a decreased glucose metabolism on the GSH system is well-known, but the effect of decreased GSH levels on the energy metabolism is unclear. The aim of the present study was to investigate the sensitivity to insulin in the mouse knockout (KO) for the modulatory subunit of the glutamate cysteine ligase (GCLM), the rate-limiting enzyme of GSH synthesis. Compared to wildtype (WT) mice, GCLM-KO mice presented with reduced basal plasma glucose and insulin levels. During an insulin tolerance test, GCLM-KO mice showed a normal fall in glycemia, indicating normal insulin secretion. However, during the recovery phase, plasma glucose levels remained lower for longer in KO mice despite normal plasma glucagon levels. This is consistent with a normal counterregulatory hormonal response but impaired mobilization of glucose from endogenous stores. Following a resident-intruder stress, during which stress hormones mobilize glucose from hepatic glycogen stores, KO mice showed a lower hyperglycemic level despite higher plasma cortisol levels when compared to WT mice. The lower hepatic glycogen levels observed in GCLM-KO mice could explain the impaired glycogen mobilization following induced hypoglycemia. Altogether, our results indicate that reduced liver glycogen availability, as observed in GCLM-KO mice, could be at the origin of their lower basal and challenged glycemia. Further studies will be necessary to understand how a GSH deficit, typically observed in GCLM-KO mice, leads to a deficit in liver glycogen storage.

## Introduction

Deficits in glutathione, a major regulator of reactive oxygen species (ROS) levels, have been observed in several neurodegenerative disorders including Alzheimer's, Parkinson's, and Huntington's diseases (For reviews see Dringen and Hirrlinger, 2003; Ballatori et al., 2009), as well as in psychiatric illnesses, such as major depressive and bipolar disorders (Gawryluk et al., 2011), and schizophrenia (Yao et al., 2006; Do et al., 2009a,b; Gawryluk et al., 2011). Mental illnesses have repeatedly been associated with the metabolic syndrome (For reviews see Henderson et al., 2015; Vancampfort et al., 2015; Xu et al., 2015), and excess levels of ROS may contribute to the development of those pathologies that have reached epidemic proportions, such as atherosclerosis and Diabetes Mellitus (For a review see Santilli et al., 2015). Therefore, understanding the impact of a chronic glutathione (GSH) deficit on energy metabolism has important clinical implications.

Glucose serves as the principal source of energy in the body. This monosaccharide can be metabolized via two pathways, the glycolysis and the pentose phosphate pathway. Glucose metabolism through the glycolysis pathway is usually followed by the tricarboxylic acid cycle yielding 30 to 36 ATP per glucose (For a review on energy metabolism see Allaman and Magistretti, 2013). Side products of this important energy-generating pathway are reactive oxygen species (For a review see Quijano et al., 2015). ROS are free radical atoms or molecules with an unpaired electron that renders them highly reactive.

Free radicals are essential in several biochemical processes including the regulation of insulin sensitivity and glucose homeostasis (For a review see Bisbal et al., 2010). However, if ROS levels become too high, they can induce significant damage to proteins, membranes and DNA (Halliwell and Chirico, 1993; Halliwell, 1999). The major cellular antioxidant and redox regulator in living cells is the tripeptide glutathione (Orlowski and Karkowsky, 1976; Meister and Anderson, 1983; Dringen, 2000; Hammond et al., 2001). GSH is needed for the reduction of reactive glycolysis by-products. It is also known that low glucose availability is accompanied by severe redox imbalance, partially due to the fact that the metabolism of glucose through the pentose phosphate pathway produces the NADPH necessary to maintain a proper GSH/GSSG redox balance (Pias and Aw, 2002; Tang et al., 2015). Thus, there is a tight link between the GSH system and glucose metabolism.

However, the way a primary GSH deficit can impact on glucose metabolic pathways remains unclear. The effects of oxidative stress and elevated free radicals on glucose metabolism have so far mainly been studied in the context of obesity and diabetes. These studies have demonstrated that high-fat diet and obesity lead to excessive production of ROS, which in turn contribute to insulin resistance (for reviews see Bashan et al., 2009; Bisbal et al., 2010). Moreover, a lower GSH/GSSG ratio and associated oxidative stress have been demonstrated to precede an increase in insulin resistance and impairment in glucose homeostasis (Paolisso et al., 1992; Nwose et al., 2006). In contrast, a study using GSH peroxidase knockout mice, which are characterized by redox imbalance and increased ROS levels, resulted in enhanced insulin sensitivity in these animals (Loh et al., 2009). The contribution of a GSH dysregulation in insulin resistance remains unclear.

A valuable model to study the effect of a chronic GSH deficit and the consequent chronic vulnerability to oxidative stress is the mouse knockout (KO) for the modulatory subunit of the glutamate cysteine ligase (GCLM), the rate-limiting enzyme of GSH synthesis (Yang et al., 2002; Lavoie et al., 2009; Steullet et al., 2010). These mice present with severe and chronic GSH deficit of 80% and more in liver, lung, kidney, pancreas, and plasma (Yang et al., 2002; McConnachie et al., 2007), as well as in brain (McConnachie et al., 2007; Steullet et al., 2010) and brain cells (Giordano et al., 2006; Lavoie et al., 2009). GCLM-KO mice also show increased oxidative stress markers levels (Kendig et al., 2011). Kendig et al. (2011) have demonstrated that GCLM-KO mice fed a high fat diet, are protected against the development of diet-induced obesity, glucose intolerance, and insulin resistance (as assessed by the HOMA-IR index, i.e., the product of fasted insulinemia X glycemia). Under normal chow feeding, GCLM-KO mice showed a normal HOMA-IR index, suggesting normal insulin sensitivity although this was not formally confirmed with tests assessing insulin sensitivity.

The present study therefore aimed to investigate the sensitivity to insulin in the GCLM-KO mouse. We hypothesized that under normal chow feeding, GCLM-KO mice would present with normal insulin sensitivity. Basal plasma glucose and insulin were measured, and insulin tolerance tests were performed. Based on the results obtained, exploratory experiments were conducting during which the counterregulatory hormone glucagon was measured as well and hepatic glycogen stocks. The glucose response to an acute resident-intruder stress was also assessed.

## Materials and methods

### Animals

GCLM-KO mice, back crossed with C57BL/6J mice over more than 10 generations, were kindly provided by Timothy P. Dalton and Ying Chen (Center for Environmental Genetics, Cincinnati, OH, USA; Yang et al., 2002). Male mice used for the present study were bred in the local animal facility under normal 12:12-h light/dark cycle. Mice were gently handled daily for 1 week prior to experiments in order to habituate them to manipulation and minimize the stress induced by handling/manipulation during injections and blood collections. All experiments were performed in accordance with the guidelines outlined in the *Guide for the Care and Use of Laboratory Animals* (National Research Council) and were approved by the Consumer and Veterinary Affairs Services of the Canton Vaud, Switzerland.

### Material

Unless otherwise stated, all chemicals were purchased from Sigma-Aldrich (St-Louis, MO, USA).

### Methods

Before all experiments, WT and KO mice were single-housed overnight for 16 h and food-restricted during the last 4 h in order to reach a stable glycemic state. Samples were collected during the animals' light phase between 12:00 and 14:00 h for all experiments.

#### Basal plasma glucose levels

Blood samples were obtained from tail-tip bleedings for immediate glycemia measurements with a glucometer (Ascensia Breeze2, Bayer AG, Leverkusen, Germany).

#### Basal plasma insulin levels

With the tail nick procedure, blood was collected with Microvette capillary tubes EDTA-2Na (Sarstedt, Nümbrecht, Germany). Blood was then immediately centrifuged (4°C, 10000 rpm, 15 min) and the plasma was frozen at −20°C until measurements. Insulin levels were quantified with a commercially available Insulin enzyme immunoassay kit (Alpco Immunoassays, Salem, NH, USA).

#### Insulin tolerance test

Mice were i.p.-injected with insulin (0.5 U/kg, diluted in BSA 0.5%; Actrapid, Novo Nordisk Pharma SA, Küsnacht, Switzerland) at around 13:00 (corresponding to 4-h fasting). Blood samples were obtained from tail-tip bleedings at the time of injection (time = 0) and 15, 30, 60, 90, and 120 min after injection. Plasma glucose levels were measured with a glucometer.

#### Plasma glucagon levels

Since large blood quantities (at least 100 μl of plasma) were necessary for glucagon measurements, animals were decapitated and trunk blood was collected with Microvette capillary tubes EDTA-2Na, to which Aprotinin was added, and was immediately centrifuged (4°C, 10000 rpm, 15 min). Plasma extracted was immediately frozen at −80°C and subsequently unfrozen for glucagon levels measurements with the Glucagon enzyme immunoassay kit (Alpco Immunoassays, Salem, NH, USA).

#### Hepatic glycogen levels

Mice were decapitated. The liver was rapidly extracted, immediately frozen on carbon dioxide ice and then kept at −80°C. For glycogen measurements, frozen samples were placed into Eppendorf tubes and weighed before NaOH 0.1 M was added to stop enzyme activity. Samples were homogenized on ice and a 50−μl aliquot was used to measure the protein content using the BCA protein assay reagent kit (Pierce, Rockford, IL, USA). Tubes were then centrifuged at 14000 g for 10 min and the supernatant was used for glycogen dosage following a previously described procedure (Allaman et al., 2010). In a first 100-μl aliquot, 300 μl of sodium-acetate buffer (0.1 M, pH 4.6) was added. In the second one, 300 μl of the same buffer containing 1% (v/v) of amyloglucosidase (10 mg/ml; Roche Diagnostics, Rothkreuz, Switzerland) was added. Aliquots were incubated at room temperature (RT) for 30 min. Then, 2 ml of Tris-HCl buffer (0.1M; pH 8.1; MgCl_2_ 3.3mM, ATP 0.2mM, NADP 30μM, containing 0.7 U/ml of hexokinase, and 0.35 U/ml of glucose 6-phosphate dehydrogenase (Roche Diagnostics)) were added, and the mixture was incubated at RT for 30 min. Fluorescence associated with the NADPH formed was then read on a fluorimeter (excitation: 340 nm; emission: 450 nm) after calibration with an appropriate standard curve using glucose as standard. The first aliquot gives the sum of glucose and glucose 6-phosphate, and the second gives the sum of glycogen, glucose, and glucose-6-phosphate; the amount of glycogen was determined by subtracting the result obtained from the first aliquot from the result obtained from the second aliquot. Results are presented in nmol glycogen per mg of protein, one mole of glycogen corresponding to one mole of glycosyl units originating from glycogen.

#### Resident-intruder stress

An adapted version of the resident-intruder paradigm (Martinez et al., 1998; Heinrichs and Koob, 2005) was used to induce stress 1 month after the ITT. For the stress procedure, a weight-matched white OF1 (Charles River, L'Arbresle, France) intruder mouse was placed into the cage of the black WT or GCLM-KO resident for a period of 30 min. Plasma glucose levels were measured immediately before and after the stress.

Plasma corticosterone (CORT) levels were measured before the resident-intruder stress, immediately after the stress and 60 min after to assess the hormonal response immediately after the stress and during the remission period. Between 20 and 30 μl of blood was sampled using the tail-nick procedure and collected with a lithium-heparin coated capillary tube (Microvette CB 300, Sarstedt, Nuembrecht, Germany). Blood samples were centrifuged (4°C, 4000 rpm, 15 min) before plasma was extracted and stored at −20°C until further processing. Blood was unfrozen for CORT levels measurements using the Corticosterone enzyme immunoassay kit (Assay Designs, Ann Arbor, MI, USA).

### Statistical analyses

Statistical analyses were performed using SPSS Statistics 17.0 (Chicago, IL, USA). For comparisons between WT and KO, the *t*-test for independent samples was used. Significant probability level was set to *p* ≤ 0.05. For the ITT, because multiple measurements were taken overtime, repeated-measure ANOVAs with Time as within-subject factor and Genotype as between factor were performed. The effect of time was then assessed with paired-samples *t*-test between each time point and time = 0, and the Bonferroni correction for multiple comparisons was used. The effect of genotype was assessed with *t*-tests for independent samples at each time point, also Bonferroni-corrected for multiple comparisons. CORT levels measured 60 min after the end of stress were not distributed normally, so the non-parametric Mann–Whitney test for independent samples was used to compare between genotypes at this time point and the Wilcoxon signed-rank test was used to assess the difference within each genotype over time.

## Results

### Lower basal plasma glucose and insulin levels in GCLM-KO mice

To establish the basal experimental condition, plasma glucose and insulin levels were measured in both GCLM-KO and –WT mice.

**Figure 1:**
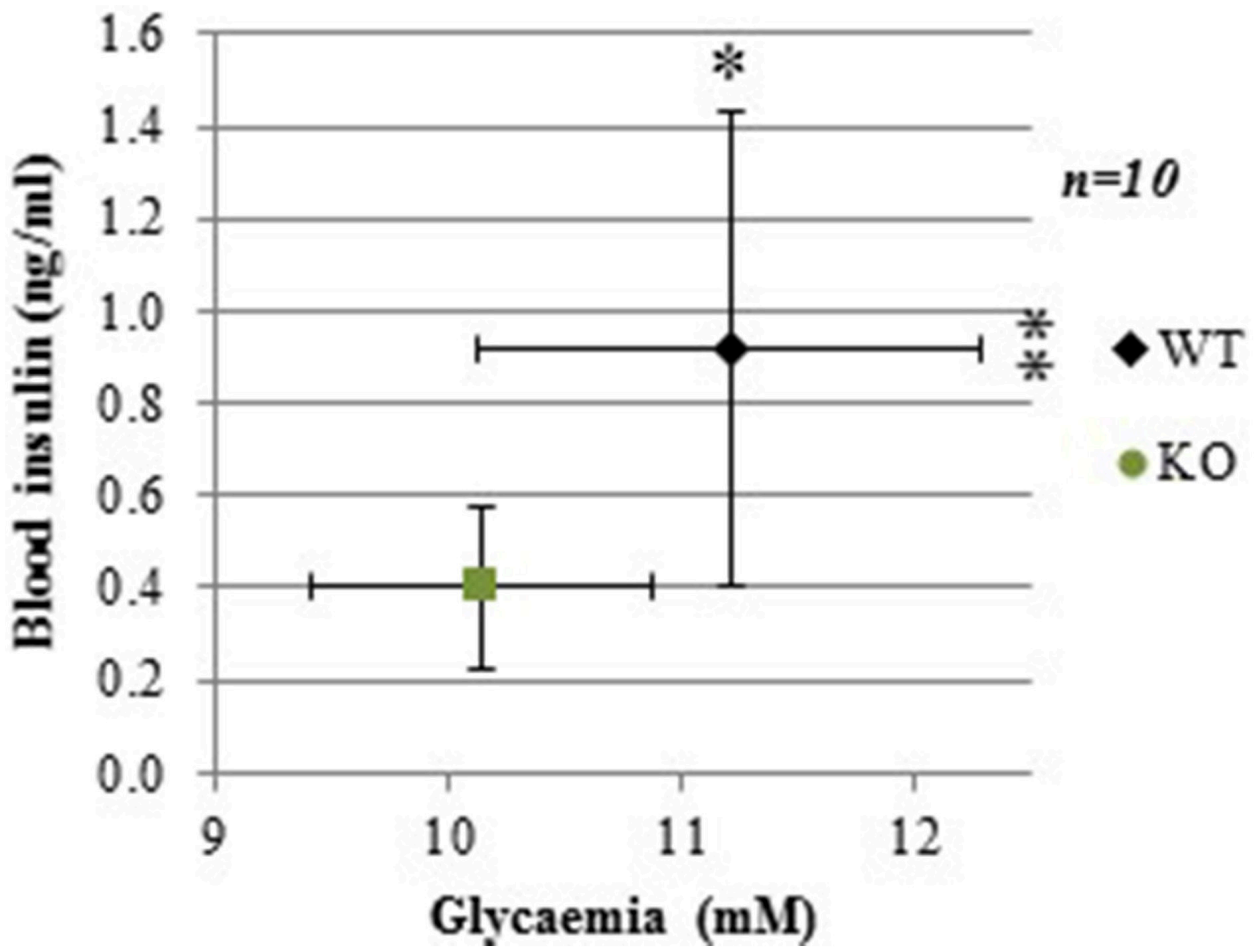
**Lower basal plasma glucose and insulin levels in GCLM-KO mice compared to WT mice**. Data are expressed as mean ± SD. ^*^p < 0.05; ^**^p < 0.01.

Following 4-h food deprivation, both plasma glucose and insulin levels were lower in GCLM-KO mice compared to WT (−9.9%, *p* = 0.015; −56.4%, *p* = 0.01, respectively; Figure 1). We reasoned that this might be due to either a primary hypoglycemia with a secondary hypoinsulinemia, and/or a primary action of insulin to more efficiently lower glycemia. In order to assess this last hypothesis, and to verify our primary hypothesis that insulin tolerance is normal in GLCM-KO mice, we assessed insulin action by an insulin tolerance test.

### Normal insulin sensitivity but delayed recovery from insulin-induced hypoglycemia in GCLM-KO mice

At the time of the insulin tolerance test (ITT) 3 months-old GLCM-KO males were lighter than WT (23.8 ± 0.4 and 26.8 ± 0.4 g respectively; −13%; *p* < 0.001).

**Figure 2:**
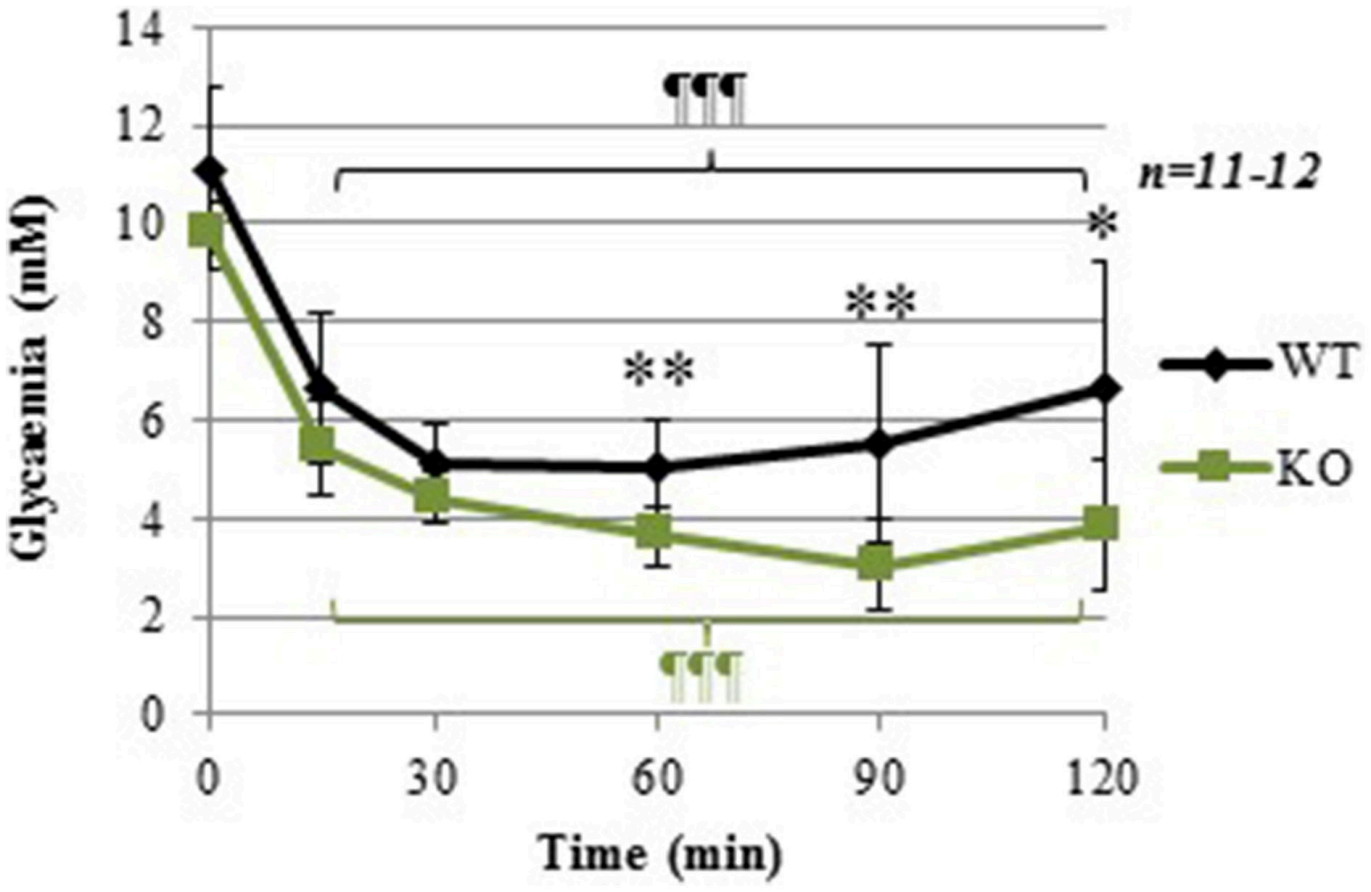
**Insulin tolerance test (ITT) in WT and GCLM-KO mice**. Data are expressed as mean ± SD. Repeated-measure ANOVA showed a significant within-subject effect of time [*F*_(5, 17)_ = 93.229; *p* < 0.001], and a significant interaction between Time and Genotype [*F*_(1)_ = 3.559; *p* = 0.008] ^*^p < 0.05; ^**^p < 0.001 vs. other genotype; ¶¶¶*p* < 0.001 vs. same genotype at time = 0.

Figure 2 shows that, as seen previously in Figure 1, basal glycemia (time = 0) tended to be lower in GCLM-KO mice (raw *p*-value = 0.023, although it did not reach statistical significance when Bonferroni-corrected for multiple measures in this test). Repeated-measure ANOVA showed a significant within-subject effect of time [*F*_(5, 17)_ = 93.229; *p* < 0.001], and a significant interaction between Time and Genotype [*F*_(1)_ = 3.559; *p* = 0.008]. Specifically, The insulin bolus decreased plasma glucose to a similar extent in both WT and GCLM-KO mice up to 30 min post-bolus, suggesting that GCLM-KO mice have normal insulin sensitivity. However, during the phase of recovery from hypoglycemia, plasma glucose levels remained significantly lower in GCLM-KO mice compared to WT at times 60 min (−27.9%; *p* = 0.001), 90 min (−44.6%; *p* = 0.001), and 120 min (−41.2%; *p* = 0.004) post-insulin-injection. These results are suggestive of an impaired counterregulation in KO mice. To investigate this hypothesis, levels of glucagon, the major participant in the counterregulation, were measured.

### Normal plasma levels of the counterregulatory hormone glucagon

**Figure 3:**
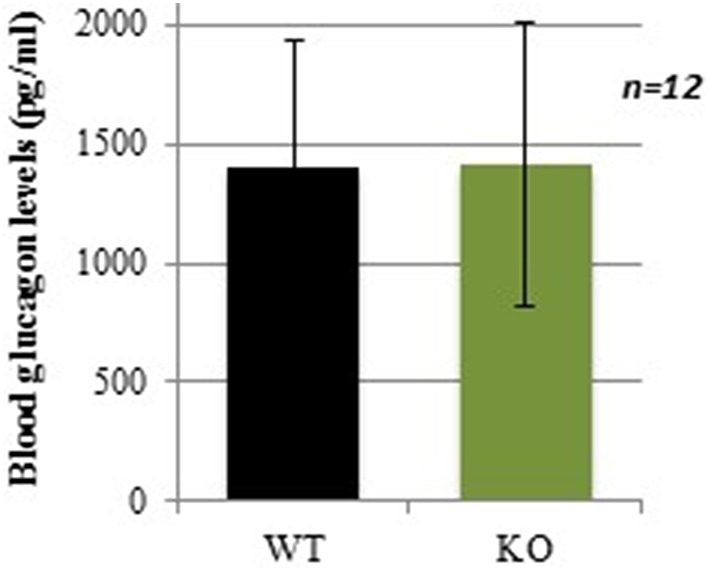
**Plasma glucagon levels in WT and GCLM-KO mice 60 min after insulin injection**. Data are expressed as mean ± SD.

Sixty minutes after insulin injection, at the time when glucose levels started to increase back to basal levels in WT mice (Figure 2), plasma glucagon levels were not significantly different between WT and KO mice (Figure 3). In absence of counterregulatory defect at the hormonal level, the delayed recovery from insulin-induced hypoglycemia could be due to an inability to mobilize glucose from endogenous stores. To assess this hypothesis, another glucose-mobilizing paradigm was tested, i.e., acute stress-induced hyperglycemia where stress hormones such as corticosterone mediate the mobilization of glucose from hepatic glycogen.

### Lower glycemic levels in response to acute social stress despite higher CORT response

**Figure 4:**
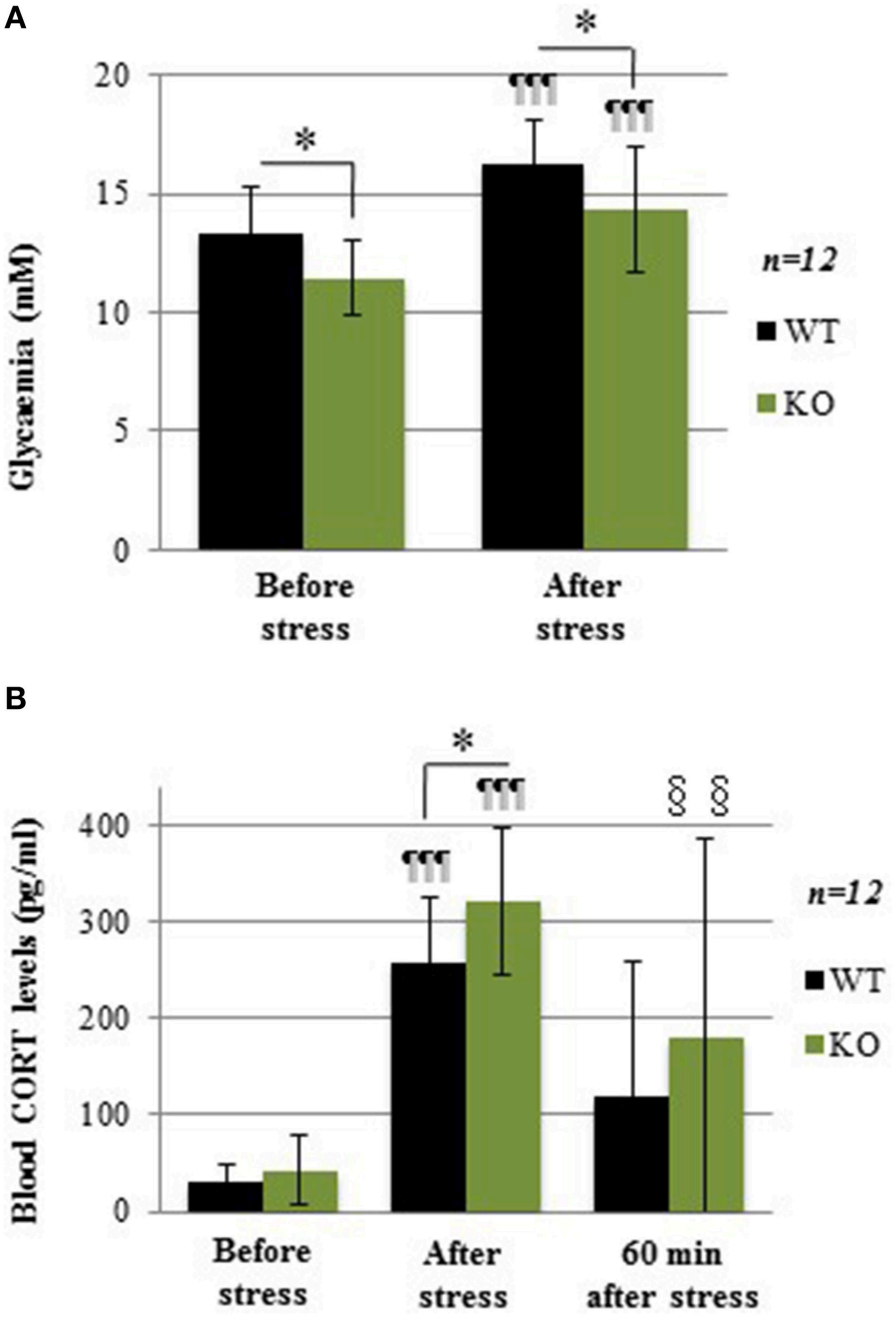
**(A)** Glycemia in WT and GCLM-KO mice before and after a 30-min resident-intruder stress. **(B)** Plasma corticosterone levels in WT and KO mice before, immediately after the stress and 60 min later. Data are expressed as mean ± SD. CORT levels were higher just after the stress period in both WT (8.4-fold increase; *p* < 0.001) and KO (7.5-fold increase; *p* < 0.001). ^*^p < 0.05 vs. other genotype; ¶¶¶*p* < 0.001 vs. same genotype immediately before stress induction (baseline). §§*p* < 0.01 vs. same genotype at baseline.

The acute social stress paradigm was used as another experimental challenge known to increase glycemia through mobilization of hepatic glycogen. Before stress induction, plasma glucose levels were lower in KO compared to WT (Figure 4A; −15.5%; *p* = 0.043). After stress, plasma glucose levels were increased in both WT (1.26-fold increase; *p* < 0.001) and KO mice (1.25-fold increase; *p* < 0.001), but the difference between the two genotypes remained the same (Figure 4A; −13.3%; *p* = 0.034). These results show that during an acute social stress, GCLM-KO mice are unable to increase glycemia at the same levels as WT mice.

Levels of the stress hormone corticosterone (CORT), which elevation mediates the stress-induced increase in glycemia, were measured before stress, immediately after stress, as well as 60 min after stress induction. At baseline, CORT levels were similar in WT and KO mice (Figure 4B). These levels were higher immediately after the stress period in both WT (8.4-fold increase; *p* < 0.001) and KO (7.5-fold increase; *p* < 0.001), with higher values in KO when compared to WT mice (+24%; *p* = 0.043). Sixty minutes after the end of the stress, CORT levels showed no more difference with baseline in WT, while they were still higher in KO (*p* = 0.004).In summary, during acute stress, although the stress-induced hormonal response was stronger in GCLM-KO mice compared to WT mice, glycemia in KO mice did not rise as high as in WT. The negative feedback control of CORT was not as robust in KO mice. Thus, overall, this result is more consistent with the hypothesis of alterations in glycogen stores rather than with an impaired ability of effector pathways to mobilize glucose from glycogen. To test this hypothesis, glycogen levels were measured.

### Lower hepatic glycogen content in GCLM-KO mice

**Figure 5:**
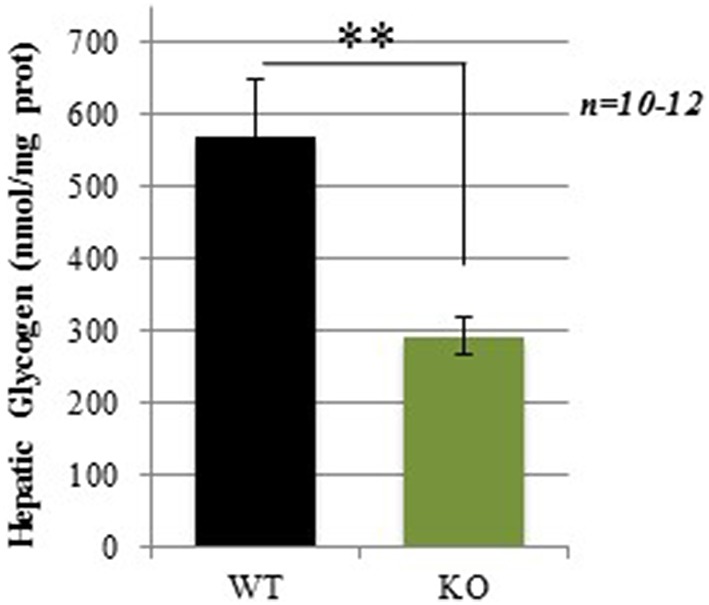
**Hepatic glycogen levels in WT and GCLM-KO mice**. Data are expressed as mean ± SD. ^**^p < 0.001.

Hepatic glycogen content was measured in mice after a 4 h fasting period. Consistent with our hypothesis, GCLM-KO mice showed a strikingly lower glycogen hepatic content compared to WT (−48.7%; *p* = 0.008; Figure 5).

## Discussion

The present study shows that GCLM-KO mice present with lower plasma glucose and insulin levels, and a reduced ability to increase plasma glucose in response to insulin-induced hypoglycemia or to acute stress. These metabolic alterations are associated with lower levels of hepatic glycogen when compared to WT mice.

During the first 30 min of the ITT, the decrease in glucose in response to insulin was comparable between the two genotypes suggesting normal insulin sensitivity. After 60 min, when glycemia started to increase back to baseline level in WT mice, it remained lower in KO mice. As the half-life of insulin is about 10 min in mice (Cresto et al., 1977), late differences in glucose concentration between groups (beyond 30 min after the insulin bolus) are not likely to reflect alterations in insulin action (Ayala et al., 2010). Thus, ITT results do not speak in favor of abnormal insulin sensitivity, as opposed to the impaired insulin sensitivity previously observed in other models of impaired GSH homeostasis (Paolisso et al., 1992; Nwose et al., 2006; Loh et al., 2009). It can be hypothesized that animals presenting chronic low antioxidant capacity have developed compensatory mechanisms to prevent such a metabolic dysregulation. Our ITT results also suggest that GCLM-KO animals display impaired counterregulatory mobilization of glucose from endogenous stores.

Compromised counterregulatory response to hypoglycemia could be due to the decrease in counterregulatory hormones released. In response to low plasma glucose levels, catecholamines, pancreatic glucagon, growth hormones, and cortisol are released, resulting in a stimulation of hepatic glycogenolysis (For a review see Amiel, 1991). Plasma levels of glucagon, the most important hormone involved in achieving recovery of glucose levels following acute hypoglycemia (Rizza et al., 1979), were similar in WT and KO mice 60 min after insulin injection (time when WT mice had started to normalize their glycemia). This indicates that the hormonal counterregulatory response to hypoglycemia is normal in KO mice. It cannot be excluded that glucagon signaling, or the activity of other hormones involved in the counterregulation may be compromised in these mice.

Compromised counterregulation might stem from an alteration in glycogen stores. Supporting this hypothesis is the observed lower hepatic glycogen levels found in KO compared to WT mice. Interestingly, in cultures of astrocytes from the GCLM-KO mice, lower basal glycogen levels and a decrease in its mobilization after an oxidative stress were observed when compared to astrocytes from WT mice (Lavoie et al., 2011). *In vivo*, already after 2 or 3 h of food removal, hepatic glycogen is usually reduced in mice (Baker and Huebotter, 1972; Seyer et al., 2013), meaning that hepatic glycogen is required for maintenance of euglycemia even shortly after food removal. Thus, lower glycogen availability and/or mobilization could be responsible for the impaired counterregulatory response during experimental hypoglycemia in KO mice. How the lack of GCLM leads to a deficiency of hepatic glycogen remains unclear.

Consistent with the role of altered hepatic glycogen stores in the counterregulatory response, the hepatic glycogen-dependent hyperglycemic response to social stress was also altered in GCLM-KO mice. When a physical or psychological stress occurs, the hypothalamic-pituitary-adrenal (HPA) axis is activated, leading to an increase in circulating glucocorticoids (For a review see Chrousos and Gold, 1992) that are known to modulate glucose metabolism. Our results clearly show that after a stress induced by the presentation of an intruder mouse for 30 min, CORT levels increased considerably in both WT and KO mice. Interestingly, the stress-induced hormonal response was stronger in GCLM-KO mice compared to WT mice indicating that the negative feedback control of CORT was less efficient in KO mice. However, the higher CORT levels in stressed KO mice did not lead to higher glycemia in these mice as compared to stressed WT mice. Therefore, even though the CORT response was supranormal, the stress-induced hyperglycemia itself appears to be compromised. This observation is in line with the hypothesis of the importance of reduced glycogen availability in the alteration of glucose homeostasis in KO mice although further studies to establish causality are warranted.

Finally, it is worth noting that this study reveals another physiological dysregulation/adaptation in GCLM-KO mice, namely an attenuated negative feedback regulation of CORT, which was also observed following a synthetic cortisol (dexamethasone) injection (unpublished observation). This observation is similar to the attenuated hormonal negative feedback response reported in patients with schizophrenia or bipolar disorder when pharmacologically challenged with dexamethasone (Mück-Šeler et al., 1999; Watson et al., 2004). It has been shown that oxidative stress induced by hyperoxia in rats led to a loss of glucocorticoid receptors in the hippocampus resulting in an elevation of the HPA activity due a decrease in the feedback regulation of the HPA axis (Kobayashi et al., 2009).

GCLM-KO mice present with chronically low GSH levels and increased oxidative stress markers levels. Knowing that the end result of glucose metabolism is accompanied by the production of reactive oxygen species, it has been postulated that adaptation toward oxidative stress in GCLM-KO mice may partly involve a constraint/limitation of glucose utilization and glycogen mobilization when an oxidative challenge is already monopolizing the GSH system (Lavoie et al., 2011). On the other hand, GCLM-KO mice present with lower weight, plasma glucose and insulin and hepatic glycogen levels compared to WT, observations consistent, among other possibilities, with a faster metabolism (Kendig et al., 2011). In this case, more ROS would be produced which would put more burden on the already deficient antioxidant system of the GCLM-KO, unless these mice switched from glycolysis (production of ROS) to the pentose phosphate pathway (generation of NADPH) as suggested by Ralser et al. (2007). In the light of the current literature, it remains unclear which energy pathway is favored by mice showing chronic oxidative stress, but it has been suggested that NADPH generation may be a more efficacious therapeutic target upstream of GSH and ROS (Ghosh et al., 2014).

## Conclusion

Our results indicate that GCLM-KO mice do not show impaired sensitivity to insulin contrary to other GSH-deficient mouse models. The GCLM-KO mice presented with reduced liver glycogen availability that could be at the origin of their lower basal and challenged glycemia, even in the present of normal levels of hyperglycemiant hormones (i.e., glucagon following insulin-induced hypoglycaemia and corticosterone following acute social stress). Further studies are warranted to assess the direct association between the deficit in GSH associated with GCLM targeted deletion and glycogen storage not only in the liver but also in other organs in both fed and fasted conditions.

## Author contributions

SL has contributed to the design of the work, as well as the acquisition, analysis, and interpretation of data for the work. She has drafted the work and approved the final version to be published. PS has contributed to the design of the work and to the interpretation of data for the work. He has critically revised the manuscript and approved the final version to be published. AK and FP have contributed to the acquisition and interpretation of data for the work. They have critically revised the manuscript and approved the final version to be published. KD and PM have contributed to the design of the work, and they have critically revised the manuscript and approved the final version to be published. All authors agree to be accountable for all aspects of the work in ensuring that questions related to the accuracy or integrity of any part of the work are appropriately investigated and resolved.

## Funding

This work was financially supported by the Swiss National Foundation No 310000-116689 and the MTR schizophrenia of the Department of Psychiatry of the Lausanne University Hospital and the EPFL.

### Conflict of interest statement

The authors declare that the research was conducted in the absence of any commercial or financial relationships that could be construed as a potential conflict of interest.

## Abbreviations

CORT: corticosterone
GCLC: glutamate cysteine ligase catalytic subunit
GCLM: glutamate cysteine ligase modulatory subunit
GSSG: oxidized glutathione
HPA: hypothalamopituitary-adrenal
ITT: Insulin tolerance test
NADPH: nicotinamide adenine dinucleotide phosphate
ROS: reactive oxygen species.
